# Comparison of continuous local anaesthetic and systemic pain treatment after axillary lymphadenectomy in breast carcinoma patients – a prospective randomized study

**DOI:** 10.2478/raon-2013-0018

**Published:** 2013-05-21

**Authors:** Branka Strazisar, Nikola Besic

**Affiliations:** 1 Department of Anaesthesiology, Institute of Oncology, Ljubljana, Ljubljana, Slovenia; 2 Department of Surgical Oncology, Institute of Oncology, Ljubljana, Ljubljana, Slovenia

**Keywords:** breast carcinoma, pain treatment, wound infusion of local anaesthetic, elastomeric pump

## Abstract

**Background:**

Acute pain after axillary lymphadenectomy is often related mainly to axillary surgery. The aim of the prospective randomized study was to find out if continuous wound infusion of local anaesthetic reduces postoperative pain, consumption of opioids and the incidence of chronic pain compared to the standard intravenous piritramide analgesia after axillary lymphadenectomy in breast carcinoma patients.

**Methods:**

Altogether 60 patients were enrolled in the prospective randomized study; half in wound infusion of local anaesthetic and half in the standard (piritramide) group.

**Results:**

In the recovery room and on the first day after surgical procedure, the wound infusion of local anaesthetic group reported less acute and chronic pain, a lower consumption of piritramide and metoclopramide, but their alertness after the surgical procedure was higher compared to the standard group.

**Conclusions:**

After axillary lymphadenectomy in breast carcinoma patients, wound infusion of local anaesthetic reduces acute pain and enables reduced opioid consumption, resulting in less postoperative sedation and a reduced need for antiemetic drugs. After wound infusion of local anaesthetic there is a statistical trend for reduction of chronic pain.

## Introduction

The sense of pain is an individual experience; it depends on pain memories and present circumstances.^1^ Breast cancer surgery can be painful, but acute pain is often related mainly to axillary surgery. Furthermore, breast surgery can be emotionally distressing.^2^ The risk of clinically meaningful acute pain was increased in patients with preoperative anxiety.^3^ Surgery and inflammation that follow the intervention, activate peripheral nociceptors in the skin, ligaments and muscles. A noxious stimulus is propagated by thin, unmyelinated C type fibres and thinly myelinated A-delta fibres to the central nervous system.^4^ During axillary lymph node dissection, the surgeon should avoid an injury of the long thoracic and thoracodorsal nerve, because the lesion of these nerves causes a muscular deficit and results in physical deformities.^5^ However, the intercostobrachial nerves are often injured or cut during axillary surgery. Already a small lesion of these nerves can cause pain.^6^ Despite a meticulous surgical technique; there is a 35%–50% risk of developing numbness, pain, or paraesthesia.

Patients who develop pain or paraesthesia are at risk of progression to chronic pain syndromes. Five years after axillary dissection, as many as 30% of patients continue to experience symptoms.^7^,^8^ Chronic pain syndrome after axillary dissection for breast carcinoma is known as post-mastectomy syndrome.^9^ The chronic pain syndrome is often a result of inadequately treated acute pain.^10^ Persistent pain can be avoided through continuous use of a sufficient level of pain medication.^9^ Perioperative analgesia has traditionally been provided by opioid analgesics. Large doses of opioids can be associated with an increased incidence of postoperative complications: respiratory depression, sedation, nausea and vomiting, pruritus and ileus.^11^,^12^ Therefore, anaesthesiologists and surgeons are increasingly turning to non-opioid analgesic techniques for managing pain during the perioperative period in order to minimize the adverse effects of analgesic medications.^12^ Local anaesthetics can improve postoperative pain management.^11^ They suppress the afferent nociceptive signal and inflammatory reaction.^13^ Local anaesthetics for postoperative analgesia are used in many fields of surgery: orthopaedics, abdominal surgery, gynaecology, urology, cardiothoracic surgery and breast cancer surgery.^14^ Continuous administration of local anaesthetics into the wound via a catheter placed directly at the end of surgery represents a simple and efficacious means to provide postoperative analgesia.^15^,^16^ At the Institute of Oncology in Ljubljana, intravenous administration of the opioid piritramide is a standard treatment of perioperative pain. Piritramide is used because it has lower frequency of side effects, *i.e.* vomiting, respiratory depression or cardiovascular instability, compared to morphine.^17^ Based on good results of our retrospective data; we wanted to prove the superiority of the continuous local anaesthetic infusion over the standard intravenous analgesia after axillary dissection in breast cancer patients. We hypothesized that a continuous infusion of the local anaesthetic through a wound catheter after axillary dissection in breast carcinoma patients reduces acute pain more effectively than the standard intravenous opioid analgesia. Our prospective randomized study was also aimed at testing if the patients with a continuous infusion of the local anaesthetic have a lower consumption of opioids, a lower need for antiemetic drugs, lower sedation, and less frequent chronic pain compared to the standard opioid based analgesia group of patients.

## Patients and methods

### Methods

Patients listed for axillary dissection because of breast cancer were screened preoperatively during the routine anaesthetic assessment. Allergies to local anaesthesia, male gender or dependence on analgesics were the criteria excluding patients from our study. Female patients undergoing breast cancer surgery with axillary dissection were randomized into a group having a wound catheter with elastomeric pump, or into the standard piritramide treatment group. From December 2010 to July 2011, a total of 60 patients were enrolled: 30 patients in the test group and 30 in the standard group.

The study was reviewed by the appropriate Ethics Committee and performed in accordance with the ethical standards laid down in an appropriate version of the 1964 Declaration of Helsinki. Our study was approved by the Institutional Review Board and conducted with the understanding and consent of the involved human subjects.

The surgical procedure was performed during general anaesthesia. Anaesthetic technique for all the patients was the same. All of them received a single prophylactic dose of antibiotic before the beginning of the surgical procedure. Patients without diabetes received 4 mg of dexamethasone. All patients were given 1 mg of granisetron in order to prevent nausea. Before the end of the surgical procedure, all patients received our standard analgesic infusion mixture, which contained 7.5 mg of piritramide, 2.5 g metamizole and 10 g of metoclopramide.

### Test group of patients

Before wound closure, 30 patients, who were randomized into the test group, received a fenestrated wound catheter, which was placed into their wound cavity near to the axillary vein and upon the whole length over the upper side of the wound. The wound catheter was fenestrated in the distal part, 15 cm in length. A bolus of 15 ml of 0.25% levo-bupivacaine was injected in the wound through the catheter immediately after wound closure. Soon after the administration of bolus, the elastomeric pump was connected to the wound catheter.

Surgical drains and the fenestrated catheter were clamped for five minutes after the administration of levo-bupivacaine in order to enable the absorption of levo-bupivacaine from the bolus. After five minutes, clamps on surgical drains were released in order to evacuate seroma. After another 15 minutes, the clamp on fenestrated catheter was also released, enabling a continuous infusion of levo-bupivacaine. The amount of the local anaesthetic in the elastomeric pump was 100 mL at the flow rate of 2 mL per hour. The catheter was removed 50 hours later, when the elastomeric pump reservoir was empty.

### Control group of patients

The control patients, *i.e*. those who received the standard intravenous piritramide treatment, were on a continuous intravenous infusion with piritramide (30 mg), metoclopramide (20 mg) and metamizole (2.5 g) in total amount of 100 mL of saline until the next morning. The rate of infusion was between 3 mL/h and 6 mL/h. The nursing staffs were instructed to maintain the lowest rate of drip infusion, which relieved the patient of her pain. The volume of infusion has been registered.

Both groups of patients could get the intravenous bolus of a rescue analgesic or an antiemetic drug whenever needed. In a case of severe pain, the patient received a bolus of 3 mg of piritramide. A total consumption of all intravenous drugs used during the first 24 hours after the surgical procedure was registered.

### Pain measurement

Pain was measured using a standard visual analogue scale (VAS) score in ranging from 0 to 10. The first measurement was made in the recovery room. Thereafter, pain was measured three, six and nine hours after the surgical procedure. Over the next two days, pain was measured every eight hours. Six hours after the surgical procedure, we measured their alertness using the Observer’s Assessment of Alertness/Sedation Scale (OAA/S Scale) in a composite way.^18^ The OAA/S Scale score is composed of four categories: 1) responsiveness, 2) speech, 3) facial expression, and 4) eyes. The composite score ranges from 1 (deep sleep) to 5 (alert). The result is the lowest level observed by the nurse in any one of the four component scores.

In case of nausea or vomiting, the patient received a bolus of an antiemetic drug, the first one being metoclopramide. If no relief was achieved, 1 mg of granisetron or, with persistent nausea, 1.25 mg of droperidol was administered intravenously. On the first postoperative day, all patients received analgesics in the form of tablets. They were administered 100 mg of diclofenac, a combination of paracetamol and tramadol and, in case of nausea, an antiemetic drug. The consumption of drugs during hospitalization was registered.

After surgical procedure the majority of patients had adjuvant treatment: chemotherapy (67%), irradiation (63%) and/or hormone therapy (82%).

All complications (inflammation, hematoma and others) were recorded. Three months after the surgical procedure the patients were asked about pain in the postoperative area or the upper extremities. All patients were examined and asked about neuropathic pain in the axilla, shoulder, arm or chest wall six months after the surgical procedure.

### Statistical analysis

The student t-test or the Mann-Whitney U test was conducted according to the data distribution. The association between categorical variables was tested by chi^2^ or Fisher’s exact test, as appropriate. All comparisons were two-sided and a p-value of ≤ 0.05 was considered statistically significant. Statistical packages PASW 18 (SPSS Inc., Chicago, IL, USA) and R 2.11.1 (R Foundation for Statistical Computing, Vienna, Austria) were used for the analysis.

## Results

The mean age of patients was 60 years (range 30–84), height 163 cm (range 150–176), weight 73 kg (range 43–114), and body mass index (BMI) 27.4 (range 15.4–41.4). There were no significant differences between both study groups either in BMI, American Society of Anaesthesiology (ASA) score, comorbidities (Table 1), length of surgical procedure, extent of lymph node dissection, volume of seroma or number of punctions of seroma (Table 2).

All lymphadenectomies were performed by eleven experienced surgeons. There were no significant differences between various surgeons either in VAS score during hospital stay (p = 0.66) or in rate of chronic pain after six months (p = 0.32).

### Acute pain

**FIGURE 1:**
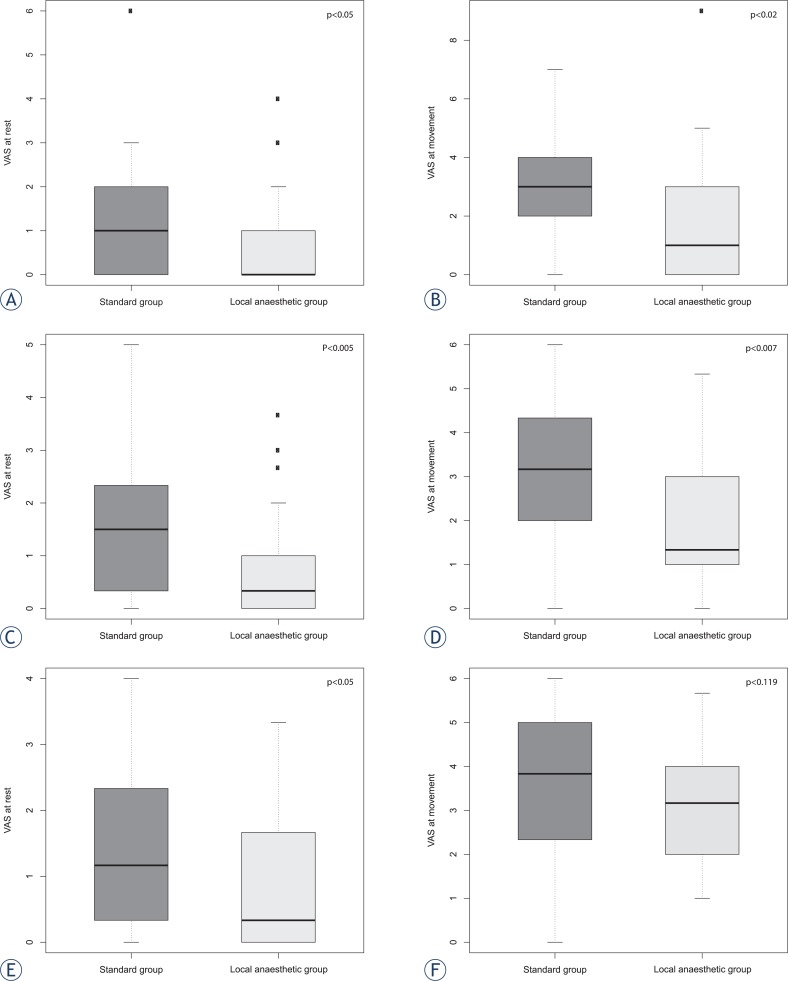
Visual analogue scale (VAS) scores at rest and at movement in the recovery room (A, B) on the day of the surgery (C, D) on the first postoperative day (E, F).

Figure 1 shows VAS scores at rest and during movement in the recovery room on the day of the surgery and on the first postoperative day. Data about postoperative pain in VAS scores are presented in Table 3.

### Opioid consumption

**FIGURE 2:**
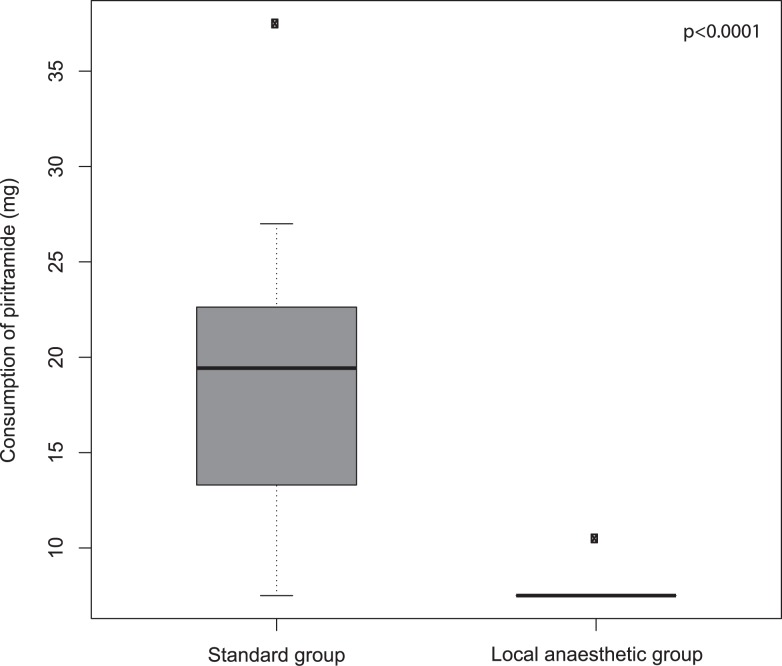
Consumption of piritramide during the first 24 hours after the surgical procedure in the local anaesthetic group and in the standard group.

Consumption of piritramide during the first 24 hours after the surgical procedure was smaller in the test group compared to the control group (p < 0.0001) (Figure 2). Alertness, as measured six hours after the surgical procedure, was higher in the test group compared to the test group (p = 0.001).

### Nausea

**FIGURE 3:**
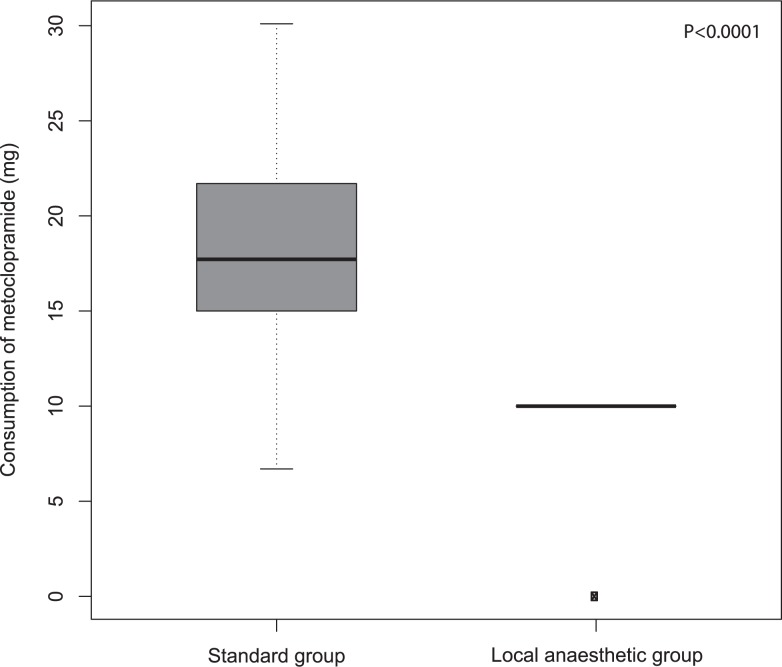
Consumption of metoclopramide during the first 24 after the surgical procedure in the local anaesthetic group and in the standard group.

Patients in the test group reported less nausea than patients in the standard group. Consumption of metoclopramide during the first 24 hours (Figure 3) after the surgical procedure was also smaller in the test group compared to the standard group (p < 0.0001).

### Complications

No local signs of infection were observed in the area, where the wound catheter was inserted. All microbiological samples taken were negative. There were no significant differences in the complications following the surgical procedure between the two groups. Altogether three patients (5%) underwent another surgical procedure because of haematoma: two cases from the test group and one case from the standard group. Inflammation after the surgical procedure occurred in nine cases (15%): five cases (17%) in the test group and four cases (13%) in the standard group.

### Hospital stay

The average postoperative hospital stay was 1.7 days. There was no significant difference in the duration of the hospital stay between the two groups.

### Late complications (three and six months after lymphadenectomy)

Questionnaire about pain was answered by patients three months after lymphadenectomy. Pain was reported by 17% and 50% of patients from test and control group (p = 0.01), respectively. Six months after surgical procedure patients from test and control group had neuropathic chronic pain in 20% and 40% (p = 0.09), respectively. Oedema of the arm was present in 21 patients, 10 from the control group and in 11 from the test group. Limited shoulder movement was present in 14 patients (23.3%), 7 from each group. Chronic pain after 6 month was present in 18 patients (30%). There were no differences in both arms of our study in frequency of chronic pain after adjuvant treatment (Table 2).

## Discussion

Axillary lymph node dissection is a standard surgical procedure in case of positive lymph nodes in breast cancer or melanoma.^19^–^21^ Unfortunately, it can cause long-term morbidities: chronic postoperative pain, limited shoulder movement and/or lymphoedema.^5^,^6^ Our present prospective randomized study showed that acute pain following breast cancer surgery and axillary lymph node dissection was less frequent and intense after a continuous wound infusion of local anaesthetic compared to a systemic intravenous analgesia with opioids. To our knowledge, there were only six studies dealing with the use of a local anaesthetic for postoperative analgesia in breast carcinoma patients.^2^,^5^,^9^,^13^,^22^,^23^ In only three of these studies^2^,^5^,^9^, a local anaesthetic was applied to the surgical wound, and only two^2^,^5^ of these three studies were designed as a prospective randomized trial. Jacobs and Morrison reported the results of a retrospective study, where the wound catheter was connected to an elastomeric pump containing local anesthetic.^9^ They found out that this type of analgesia was safe and reduced postoperative pain.^9^ Talbot *et al.* performed a prospective, double-blind, randomized, placebo controlled trial on 42 patients after a modified radical mastectomy.^2^ They did not find any difference in analgesia between the treatment group, which received levo-bupivacaine irrigation through the axillary wound drain every four hours for the first 24 hours postoperatively, and the control group, which received irrigation with normal saline.^2^ Instead of perforated catheters, they used axillary drains, which were clamped for 20 minutes every four hours following the application. Thus, a local anaesthetic was not administered continuously. Furthermore, because they did not use perforated catheters, it is questionable if the local anaesthetic irrigated the entire wound area. Our patients with a continuous infusion of local anaesthetic had a lower consumption of opioids and a reduced need for antiemetic drugs compared to the standard opioid-based analgesia group of patients. These results are in accordance with the conclusions of the majority of investigators who studied the role of local anaesthetics in postoperative pain management.^5^,^9^,^13^,^23^–^29^

In our study a cost-benefit analysis was not done. The costs of drugs were almost the same in both study arms. So, the cost of treatment with elastomeric pain pump is bigger than standard pain treatment for a price of elastomeric pain pump and perforated catheter, which is 175 Euros in our country. But, we proved that patients with a continuous infusion of local anaesthetic had a lower sedation rate compared to the standard opioid-based analgesia group of patients as a consequence of a reduced use of opioids. Almost all patients from the test group had an excellent alertness OAA/S score already on the day of the surgical procedure. Therefore, we believe that a continuous infusion of a local anaesthetic represents a very good pain management option for patients who underwent axillary lymph node dissection and are thus treated as a day case, which has already become a standard of care in some hospitals, for example in the Memorial Sloan-Kettering Cancer Centre in New York.^30^ Painless postoperative patient management and a shorter hospital stay are an essential part of a patient-friendly treatment.

Another very important issue of breast cancer treatment after axillary surgery is the incidence of chronic pain. Many factors are involved in the development of chronic pain after a surgical procedure: genetic susceptibility, psycho-social background, age and gender.^31^ Smith *et al.* identified age, marital status, employment status and housing as risk factors for post-mastectomy pain syndrome.^32^ Many studies and reviews have noted that a more severe acute postoperative pain is a risk factor for the development of chronic postoperative pain.^16^ It is well known that an infusion of local anaesthetics in the peripheral nerve sheath reduces the incidence of chronic pain after a lower limb amputation^33^, and that an epidural block with a local anaesthetic before the surgery reduces long-term post-thoracotomy pain.^34^ However, the use of a local anaesthetic immediately after the surgical procedure to prevent the post-mastectomy pain syndrome is still not sufficiently defined. Three months after lymphadenectomy, our patients on postoperative continuous infusion of local anaesthetic reported pain less often than patients on opioid-based analgesia (17% *vs*. 50%; p = 0.01).

However, six months after lymphadenectomy there was just a trend for lower pain in continuous infusion of local anaesthetic group of patients in comparison to opioid-based analgesia (20% *vs.* 40%; p = 0.09). Similar outcome on occurrence of post-mastectomy pain syndrome was reported by Fassoulaki *et al*.^13^,^22^ In their first study, the anaesthetic cream was applied locally on the skin just before and at the end of the surgical procedure, and daily thereafter for a total of four days. The incidence of pain three months after surgical procedure was 43% in the study group and 91% in the placebo group (p = 0.002).^22^ In their second study, the incidence of pain after three months was 45% in the treatment group (local anaesthetic cream, irrigation of brachial plexus and intercostal places with a local anaesthetic and gabapentin tablets) versus 82% in the placebo group (p = 0.028). They reported that chronic pain after six months in the treatment group (30%) was less common than in the placebo group (57%), but the difference was not significant. Obviously, a local anaesthetic applied at the time of a surgical procedure can affect the frequency and intensity of pain in subacute phase, but it is questionable if it can affect chronic post-mastectomy pain syndrome. In our study, the complication rate after a surgical procedure did not differ in patients treated with a continuous infusion of a local anaesthetic through the wound catheter and those who received a standard intravenous opioid analgesia. The experience of other authors who used local anaesthetics is similar. The rates of inflammation or hematomas were not higher after the use of local anaesthetics compared to the placebo or standard analgesia treatment groups.^2^,^5^,^9^,^13^,^15^,^24^–^26^,^28^,^29^,^33^,^34^ On the contrary, local anaesthetics seem to reduce the occurrence of inflammation.^35^,^36^,^37^ Like other authors, we also did not notice any toxic side effects of local anaesthetics. In our study, we used 0.25% levo-bupivacaine, which was reported as one of the safest local anesthetics.^38^

To conclude, our prospective randomized study confirmed that the application of a wound catheter with an elastomeric pump with a local anaesthetic is safe, easy and effective for reducing acute postoperative pain. Continuous infusion of a local anaesthetic into the wound reduces opioid consumption and results in less postoperative sedation and a reduced need for antiemetic drugs. The elastomeric pump is comfortable for both the patients and a nursing stuff. Thus, the patients are more alert, do not feel pain and, consequently, do not need intensive monitoring and nursing care.

## Figures and Tables

**TABLE 1:** Characteristics of patients

**Characteristic**	**Subgroup**	**Local anaesthetic group**	**Standard group**	**p-value**
Number of patients		30	30	-
Age (years) – mean		57.4	62.9	0.79
Height (m) - mean		1.62	1.637	0.43
Weight (kg) - mean		72.7	73.8	0.76
Body Mass Index (kg/m^2^) - mean		27.68	27.43	0.86
ASA score	1	7	3	0. 46
	2	22	25	
	3	1	2	
Associated diseases		19	24	0.25
Diabetes mellitus		4	8	0.33
Fibromyalgia		0	1	-
Rheumatoid artritis		0	0	-
Depression		1	4	0.35
Side of breast carcinoma	Left	18	20	0.79
	Right	12	10	
Type of invasive carcinoma	Ductal	28	27	1.00
	Lobular	2	3	
Gradus	I	0	1	1.00
	II	11	11	
	III	19	18	
Metastatic lymph nodes – mean		5.2	5.3	0.37
Resected lymph nodes – mean		17.3	19.3	0.15
Hormone receptors positive		24	26	0.73
HER-2 positive		5	6	1.00

**TABLE 2:** Treatment of patients and chronic pain after adjuvant therapy

	**Subgroup**	**Local anaesthetic group**	**Standard group**	**p-value**
Surgical procedure	Modified radical mastectomy	16	19	0.29
	Axillary lymph node dissection	13	8	
	Quadrantectomy with axillary lymph node dissection	1	3	
Median duration of surgical procedure (minutes)		72	62	0.18
Extent of axillary dissection	Three levels	29	28	0.61
	Two lewels	1	2	
Number of patients with punction of seroma		19	23	0.42
Seroma - mean volume per patient (mL)		206	353	0.24
Seroma - cumulative volume (mL)		6175	10601	0.07
Number of punctions for seroma - mean		1.77	2.83	0.15
Neoadjuvant chemotherapy	Yes	6	4	0.73
	No	24	26	
Postoperative chemotherapy	Yes	22	18	0.21
	No	8	12	
Postoperative radiotherapy	Yes	19	19	1.00
	No	11	11	
Hormone therapy	Yes	24	25	1.00
	No	6	5	
Pain 6 month after chemotherapy (N=40)	Yes	5	6	0.50
	No	17	12	
Pain 6 month after radiotherapy (N=38)	Yes	5	8	0.30
	No	14	11	
Pain after 6 months on hormone therapy (N=49)	Yes	6	10	0.36
	No	18	15	

**TABLE 3:** Pain, consumption of drugs and alertness in local anaesthetic group and standard group of patients

		**Local anaesthetic group**	**Standard group**	**p-value**
VAS in recovery room	at rest	0.0	1.0	0.05
	at movement	1.0	3.0	0.02
VAS pain on surgery day	at rest	0.3	1.5	0.005
	at movement	1.3	3.2	0.007
VAS pain on first pooperative day	at rest	0.3	1.2	0.05
	at movement	3.2	3.8	0.119
Opioid consumption during first 24 hours (mg)		7.5	19.4	< 0.0001
Metamizol consumption during first 24 hours (g)		2.5	3.5	< 0.0001
Metoclopramide consumption during first 24 hours (mg)		10	17.7	< 0.0001
Tramadol/paracetamol consumption during first three days (tablets)		4	6	0.035
Diclofenac consumption during first three days (mg)		200	200	0.13
Alertness OAA/S six hours after surgery		5.0	4.5	0.001
Pain after three months		5	15	0.01

Data is presented as the median value or number.

VAS = visual analogue scale; OAA/S = Observer’s Assessment of Alertness/Sedation
